# Bioactive glass added to autogenous bone graft in maxillary sinus augmentation: a prospective histomorphometric, immunohistochemical, and bone graft resorption assessment

**DOI:** 10.1590/1678-7757-2017-0296

**Published:** 2018-05-07

**Authors:** Juliana Dreyer MENEZES, Rodrigo dos Santos PEREIRA, João Paulo BONARDI, Geraldo Luiz GRIZA, Roberta OKAMOTO, Eduardo HOCHULI-VIEIRA

**Affiliations:** 1 Univ. Estadual Paulista , Faculdade de Odontologia de Araraquara , Araraquara , São Paulo , Brasil .; 2 Univ. Estadual Paulista , Faculdade de Odontologia de Araçatuba , Araçatuba , São Paulo , Brasil .; 3 Univ. Estadual Paulista , Faculdade de Odontologia de Araçatuba , Departamento de Ciências Básicas , Araçatuba , São Paulo , Brasil .; 4 Univ. Estadual Paulista , Faculdade de Odontologia de Araraquara , Departamento de Cirurgia e Diagnóstico , Araraquara , São Paulo , Brasil .

**Keywords:** Maxillary sinus, Bone graft, Immunohistochemistry

## Abstract

**Objective:**

The aim of this study was to compare the bone resorption rate, histomorphometry and immunohistochemical findings of bioactive glass (Biogran; Biomet, Warsaw, IN, USA) mixed with autogenous bone grafts (1:1) and autogenous bone graft isolate in maxillary sinus elevation surgery.

**Material and Methods:**

A total of 9 maxillary sinuses were grafted with Biogran with autogenous bone graft (group 1) and 12 were mixed with autogenous bone graft (group 2). Postoperative cone beam computed tomography (CBCT) was used to measure the initial graft volume after 15 days (T1), and 6 months later, another CBCT scan was performed to evaluate the final graft volume (T2) and determine the graft resorption rate. The resorption outcomes were 37.9%±18.9% in group 1 and 45.7%±18.5% in group 2 (P=0.82). After 6 months, biopsies were obtained concurrent with the placement of dental implants; these implants were subjected to histomorphometric analysis and immunohistochemical analysis for tartrate-resistant acid phosphatase (TRAP).

**Results:**

The average bone formation in group 1 was 36.6%±12.9 in the pristine bone region, 33.2%±13.3 in the intermediate region, and 45.8%±13.8 in the apical region; in group 2, the values were 34.4%±14.4, 35.0%±13.9, and 42.0%±16.6 of new bone formation in the pristine bone, intermediate, and apical regions, respectively. Immunostaining for TRAP showed poor clastic activity in both groups, which can indicate that those were in the remodeling phase.

**Conclusions:**

The similarity between the groups in the formation and maintenance of the graft volume after 6 months suggests that the bioactive glass mixed with autogenous bone (1:1) can be used safely as a bone substitute for the maxillary sinus lift.

## Introduction

Rehabilitation of fully or partially edentulous patients in the posterior maxillary bone region is frequently limited by bone quality and quantity, often requiring grafting techniques, especially when implant-supported prostheses are planned ^7^ ^,^ ^24^ . Maxillary sinus bone augmentation using bone substitutes has been used as an alternative to reestablish the bone height of these regions ^21^ .

The autogenous bone graft has osteoconductive, osteoinductive, and osteogenic characteristics and, due to this, it is the most favorable material for maxillary sinus lift ^17^ ^-^ ^20^ . Some authors have proposed the mixture of biomaterials to the autogenous bone graft to increase the graft volume without removing large amounts of bone from the donor sites. Besides these desirable results, this technique allows to perform the maxillary sinus bone augmentation using autogenous bone from the oral cavity under local anesthesia to add osteoinductive characteristics to the materials and improve the predictability of long-term resorption ^2^ ^,^ ^13^ ^,^ ^17^ . A study evaluated the bone formation and maturation in human maxillary sinus augmentation using ChronOS combined with autogenous bone graft in a 1:1 ratio, Bio-Oss added to autogenous bone graft in a 1:1 ratio and autogenous bone graft alone. The outcomes showed similar bone formation in the autogenous and ChronOS groups. However, the group grafted with Bio-Oss added to autogenous bone graft in a 1:1 ratio showed slow resorption of graft particles with discrepant outcomes compared with autogenous bone graft alone ^21^ .

Bioactive glass ceramic is a biomaterial characterized by its potential for osteoconduction, resistance, biocompatibility, and bioactivity, that is, the ability to bind to the tissues ^3^ ^,^ ^29^ . When implanted *in vivo* , the bioactive glass forms a layer of silica-rich gel on its surface and above this, a layer of calcium and phosphorus. The calcium and phosphorus layer are considered essential for the adhesion of collagen fibers and differentiation of osteopromising cells on the surface of the material ^12^ ^,^ ^14^ ^,^ ^23^ . The main advantages of bioactive glass are the fact that it is an absorbable synthetic material, free from risks of disease transmission or immunological responses and an aid in hemostasis ^10^ . Bioglass is in clinical use in the form of ﬁne particulate, dense blocks, scaffolds and granulates of various sizes for bone defect ﬁlling and orthopedic applications ^1^ .

The applicability of this bone substitute has been evaluated in some studies, which showed good results when it was used as a bone substitute in maxillary sinus lift procedures ^26^ ^,^ ^27^ ^,^ ^30^ .

In addition to the histological evaluation, the immunohistochemical analysis may provide a better understanding of the cellular events in the period of bone repair associated with the biomaterials, allowing the identification of specific proteins during this process. In this context, the use of immunolabeling for TRAP (tartrate-resistant acid phosphatase) allows the observation of osteoclast activity on the bone surface during the remodeling process in the graft ^3^ ^,^ ^11^ .

Few studies evaluated the clinical behavior of bioactive glass ceramic in the maxillary sinus bone augmentation, but there is a shortage of researches that evaluates the cellular behavior and its volumetric changes when associated with the autogenous bone ^3^ ^,^ ^5^ ^,^ ^15^ ^,^ ^25^ ^-^ ^27^ . Regarding these considerations, the purpose of this study was to perform the histomorphometric, immunochemistry, and volumetric analysis of the bioactive glass ceramic associated with the autogenous bone in a ratio of 1:1 in human maxillary sinus bone augmentation comparing it with autogenous graft alone.

The hypotheses of this study were:

H0 (null hypothesis)=Bioactive glass added to autogenous bone in a 1:1 ration has more new bone formation than autogenous bone alone.

H1 (alternative hypothesis)=Bioactive glass added to autogenous bone in a 1:1 ration has less new bone formation than autogenous bone alone.

## Material and methods

This prospective clinical study was performed from March 2014 to November 2015 and approved by the ethical committee (protocol No. 03416512.7.0000.5420). Patients with edentulous posterior maxillary bone regions and a bone height of less than 5 mm and those who required bone augmentation for dental implant placement were included. Patients were excluded if they presented uncontrolled systemic problems or local problems, such as uncontrolled periodontitis, a sinus pathology, or the presence of a residual root in the maxillary sinus. Smokers and patients who had received radiation treatment in the head and neck region were also excluded. Maxillary cone beam computed tomography (CBCT) was performed previously to evaluate the maxillary sinus and the bone height remaining in the maxillary floor; and a mandibular CBCT to analyze the retromandibular and symphysis regions to determine the volume of the mandible where the bone grafts were harvested, as well as the anatomical structures close to the region, such as teeth roots and mandibular canal.

Based on these parameters, twenty-one patients met the inclusion criteria and were selected for this study, with a total of 27 sinus surgeries. Two groups were created for this study: group 1, which had 14 maxillary sinuses grafted with bioactive glass (Biogran; Biomet, Warsaw, IN, USA) mixed with autogenous bone (1:1), and group 2, which had 13 maxillary sinuses grafted with autogenous bone alone. The number of the samples for each group was determined a by statistical power test conducted in the website (www.lee.dante.br ) based on previous results. No association was found between the side and the grafting material used. Randomization was performed by drawing lots to decide which sites would be grafted with each material.

All surgical procedures were performed by the same surgeon, with a strict aseptic protocol and under local anesthesia (lidocaine 2% with epinephrine 1:100,000; DFL, Taquara, RJ, Brazil). Then the autologous bone was collected from the mandibular ramus and triturated.

An incision was made over the alveolar crest of the regions to be grafted. After sub-periosteal detachment, the maxillary sinuses were accessed through the side wall. After detachment and elevation of the sinus membrane, the sinus was filled with either autogenous bone or bioactive glass mixed with autogenous bone in a 1:1 ratio. The sutures were done using 4–0 Vicryl resorbable thread (Ethicon, Johnson & Johnson, São José dos Campos, SP, Brazil).

During week 1, all patients were medicated with paracetamol 500 mg four times *per* day to reduce pain and amoxicillin 500 mg three times *per* day (both produced by EMS, São Paulo, SP, Brazil).

One patient from group 1 (unilateral graft) was infected during the post-operative period and was excluded from the research. After 6 months, when all patients were invited to the harvest of the samples and dental implant placement, two patients from group 1 (bilateral graft) and one patient from group 2 (unilateral graft) did not return. Thus, the analysis of this study included nine sinuses from group 1 and twelve from group 2.

### Histomorphometric analysis

Biopsy samples were collected at the time of dental implant placement with a 3.0x15 mm trephine bur (MK Life; Porto Alegre, RS, Brazil) and stored in a 10% formalin solution (pH 7) for 48 h. The samples were stored in a manner to guide the apical orientation, then they were washed in running water for 24 h and decalcified in an EDTA solution for 4 weeks. The solution was changed weekly. Next, the samples were embedded in paraffin following the apical orientation, sliced to a thickness of 5 µm, placed on slides, and stained with hematoxylin and eosin. The biopsies were evaluated by light microscopy, and the images were captured using the attached digital camera (JVC TK1270 Color Video Camera) in x12.5 magnification. Each biopsy was codified in three regions: pristine bone (2 mm above the upper side of the maxillary sinus floor), intermediate, and apical (2 mm below the Schneiderian membrane) regions, as recommended by Pereira, et al. ^15^ (2017). The maxillary sinus floor was considered the most cortical bone in the lower part of the samples following the bone height determined by the CBCT. New bone formation was analyzed by histometry using a grid of Merz added to the images by PowerPoint ^®^ for Mac (Microsoft ^®^ , Redmond-WA, EUA).

### Immunohistochemical analysis

Primary polyclonal goat antibodies raised against human TRAP (Santa Cruz Biotechnology; Santa Cruz, CA, USA) were used in immunohistochemical assays. A biotinylated donkey anti-goat secondary antibody (Jackson Immunoresearch Laboratories, West Grove, PA, USA) coupled with Avidin (Vector Laboratories, Burlingame, CA, USA) was used for signal amplification. The binding reaction was detected with diaminobenzidine (Sigma-Aldrich, St Louis, MO, USA), a chromogenic substrate for Avidin. The biopsies were divided into the same three regions as the histomorphometric evaluation. Data analyses were performed using a single-evaluator semi-quantitative approach, with scores of “0” indicating the absence of staining and scores of “1,” “2,” or “3” indicating low, moderate, or intense staining, respectively ^16^ .

### Volumetric analysis

**Figure 1:**
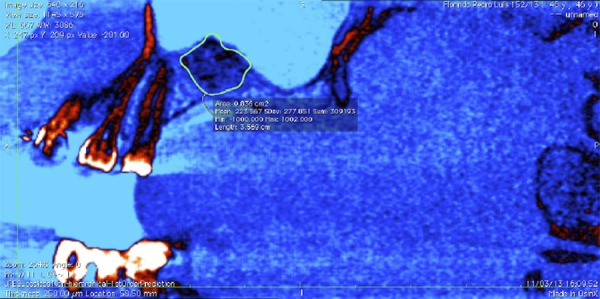
Example of an image used to measure the volume of Biogran ® graft, generated by the OsiriX software. The contour of the graft in the computed tomography slice is bounded in green, and the area is evaluated automatically by the software

The CTs were taken in two occurrences; 15 days after the first surgical procedure to determine the initial volume (T1) and after six months to determine the final volume (T2). The images were performed using an I-Cat tomography with a 0.25 mm thickness (Kavo of Brazil – Joinvile, SC, Brazil) and filed in a DICOM form. These files were evaluated by Osirix ^®^ (Osirix ^®^ Foundation, Genève, Switzerland) software, and all images were standardized to the sagittal slices (cross section). Each slice was reduced to a 1 mm distance between them using the “Reduce series” tool. The contrast and exposure were adjusted to “center level” (L=667) and “bandwidth” (W=3086) to facilitate the structures’ contour as recommended previously ^8^ ^,^ ^9^ ^,^ ^18^ . The bone graft is better visualized with the selection of image filter “flow” at the “CLUT” tool in the toolbar. The bone graft was contoured manually using the *trackpad* of a MacBook Pro (Apple – Cupertino, CA, EUA) by the tool “Pencil” (Figure 1). After the end of this procedure, the software informed the area of the graft in mm ^2^ . Each slice was filed in a TIFF (tagged image file format) in the computer’s hard drive. The bone graft volume was measured summing the area of all slices and multiplying by the height (h=distance between each slice), as recommended by Uchida, et al. ^28^ (1998) and Spin-Neto, et al. ^22^ (2013). The bone graft resorption was expressed in percentage by the equation T2-T1.

### Statistical analysis

A Shapiro-Wilk test was performed to evaluate whether samples had a normal distribution. Comparisons between groups were performed using *t* test to new bone formation and to assess the bone graft resorption (SigmaPlot 12.3 – Systat Software; San José, CA, USA). The relationship between volume changes of the augmented bone and elapsed time was evaluated by a Pearson correlation coefficient. An a priori p-value <0.05 was used for all tests.

## Results

### Histomorphometry

**Figure 2:**
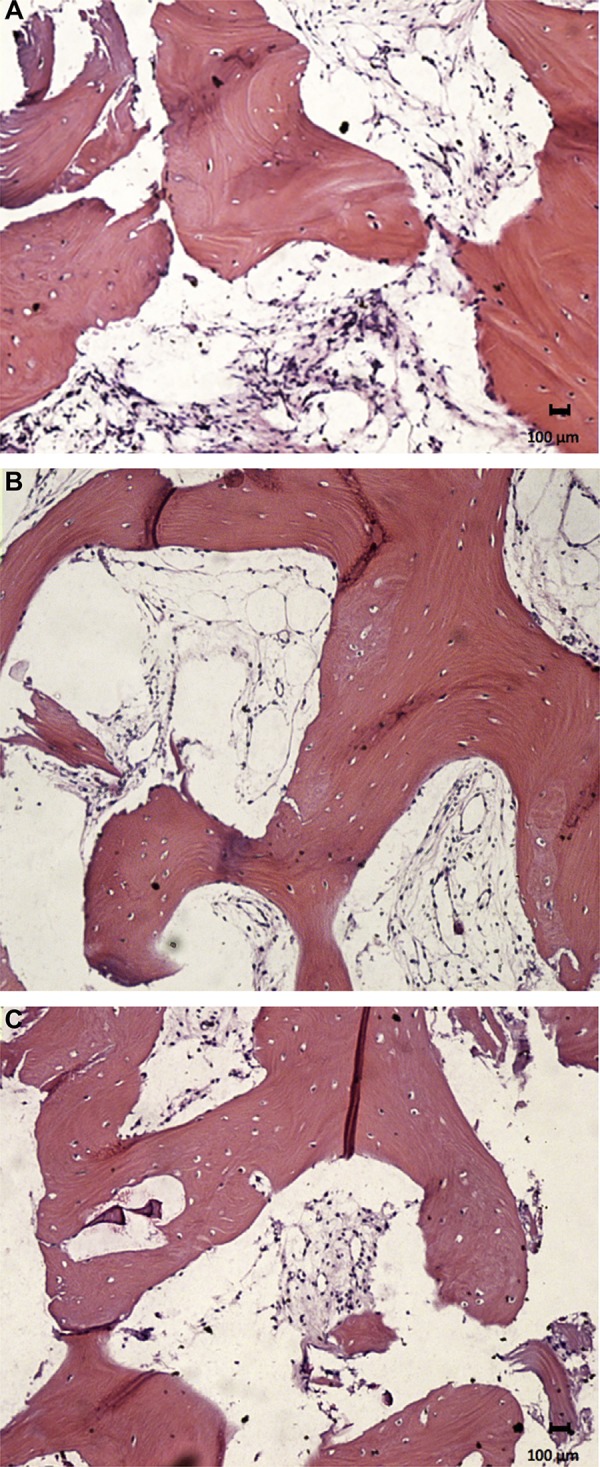
Image showing the histological section from group 1. A: Lamellar bone formation with a well cellularized connective tissue in the pristine bone region; B: Presence of lamellar bone formation and woven bone areas in the intermediate region; C: The new bone formation with an immature pattern in the apical region. Hematoxylin & eosin stain, x12.5 magnification

**Figure 3:**
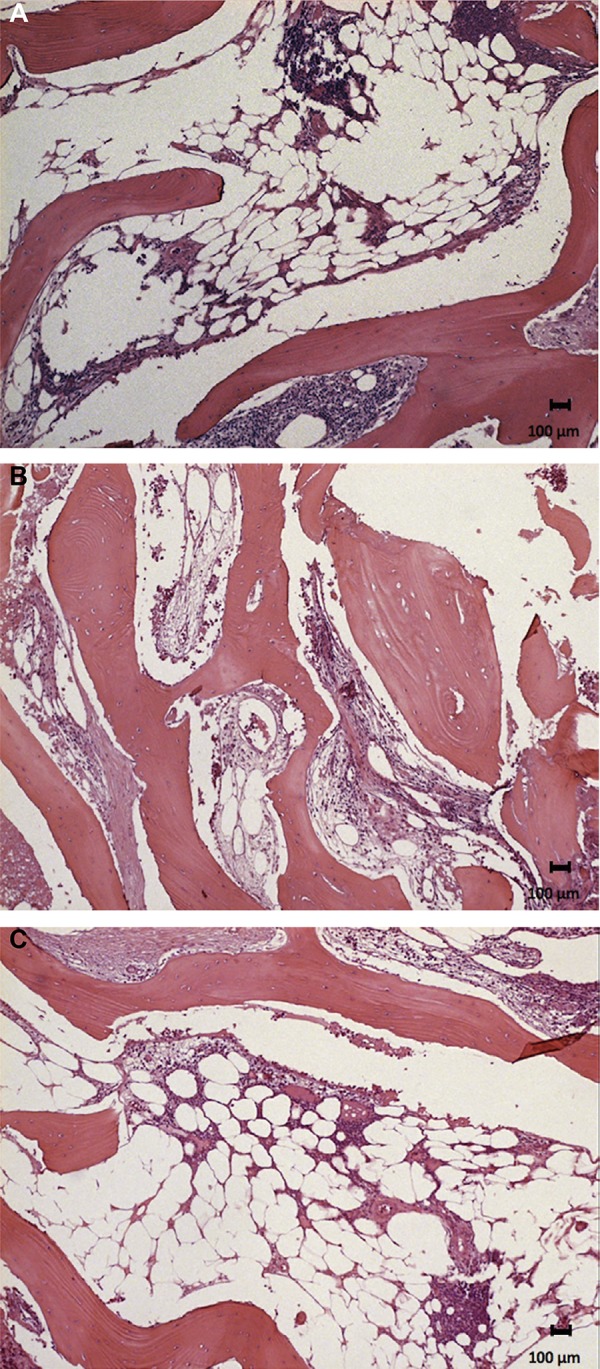
Image showing the histological section from group 2. Lamellar bone formation in a well-cellularized connective tissue in A: Pristine bone region; B: Intermediate region; C: Apical region. Hematoxylin & eosin stain, x12.5 magnification

**Figure 4:**
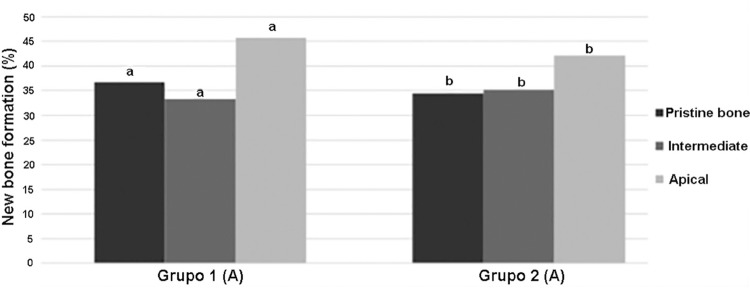
Graphic demonstrating the histomorphometric outcomes of the new bone formation after six months in the maxillary sinus augmented with Bioactive glass + autogenous bone graft 1:1 and pure autogenous bone graft. Data with the same letters (capital for each group, lower case for columns) no statistical difference was found (p<0.05)

The new bone formation for group 1 was 36.6%±12.9 in the pristine bone region, 33.2%±13.3 in the intermediate region, and 45.8%±13.8 in the apical region. The group presented areas of woven bone for all three regions evaluated, with typical trabecular bone of type IV and well-cellularized connective tissue. In the pristine bone region, the new bone formed presented lamellar formation, followed by the intermediate region with a well-cellularized matrix and immature formations. The apical region was also well-cellularized but with immature patterns and few areas of lamellar formation. (Figures 2A, 2B, and 2C). In group 2, there was 34.4%±14.4, 35.0%±13.9, and 42.0%±16.6 of new bone formation in the pristine bone, intermediate, and apical regions, respectively. After 6 months of bone repair, this group had a lamellar matrix with low areas of immature bone in all three regions evaluated. This group presented as mature, with an organized matrix and the presence of osteoblasts in the periphery (Figures 3A, 3B, and 3C). No statistical significance in bone formation was found between the two groups, as well as among the three regions within each group (p>0.05) (Table 1) (Figure 4). After these results, the hypothesis h0 was accepted and h1 was denied.

**Table 1:** Histometric outcomes of new bone formation after six months of bone repair in human maxillary sinus with the two biomaterials evaluated

Regions	Group 1 (%) A	Group 2 (%) A
Pristine bone	36.6 12.9 ^a^	34.4±14.4 ^b^
Intermediate	33.2±13.3 ^a^	35.0±13.9 ^b^
Apical	45.8±13.8 ^a^	42.0±16.6 ^b^

Data with the same letters (capital for columns, lower for lines) no statistical difference was found (p<0.05)

### Immunochemistry

**Figure 5:**
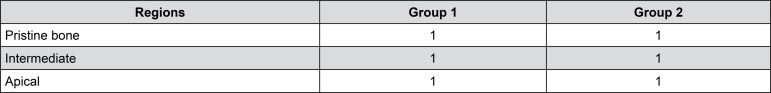
Score assigned to TRAP immunostained after six months of bone repair in both groups for each region of samples

**Figure 6:**
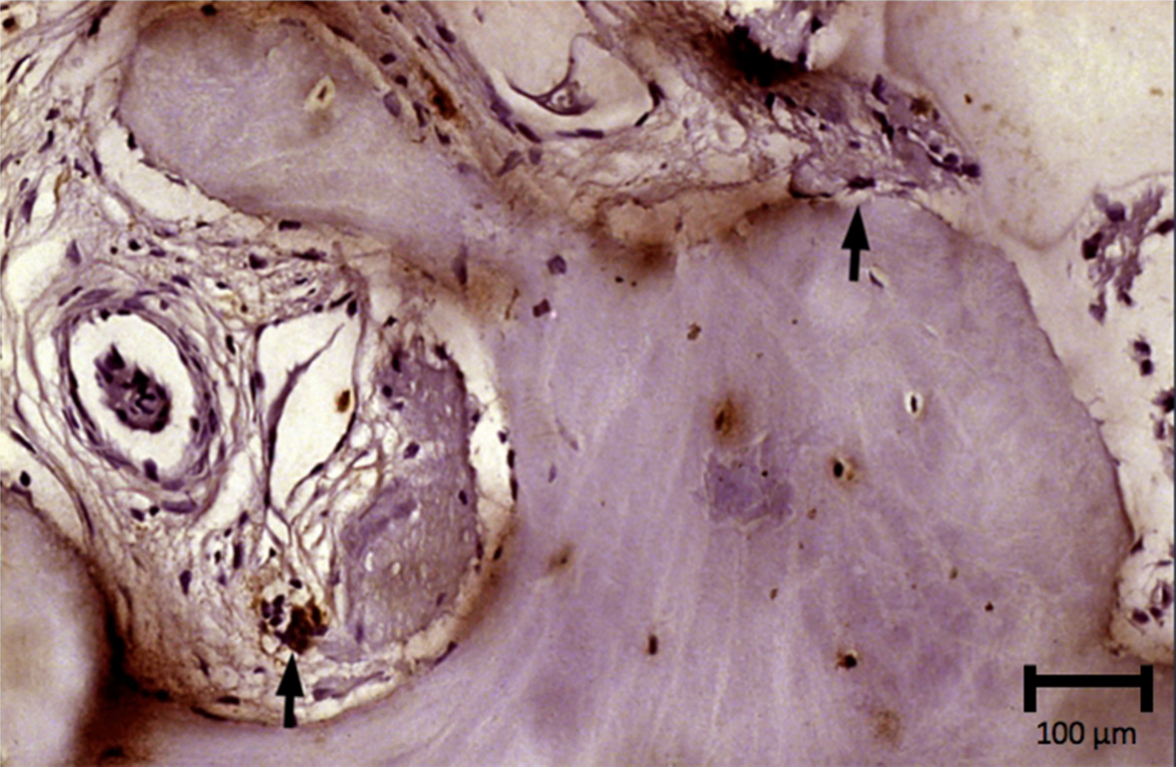
Histological section showing positive immunolabeling in cells for TRAP (→) in group 1. Harri’s hematoxylin stain, x25 magnification

**Figure 7:**
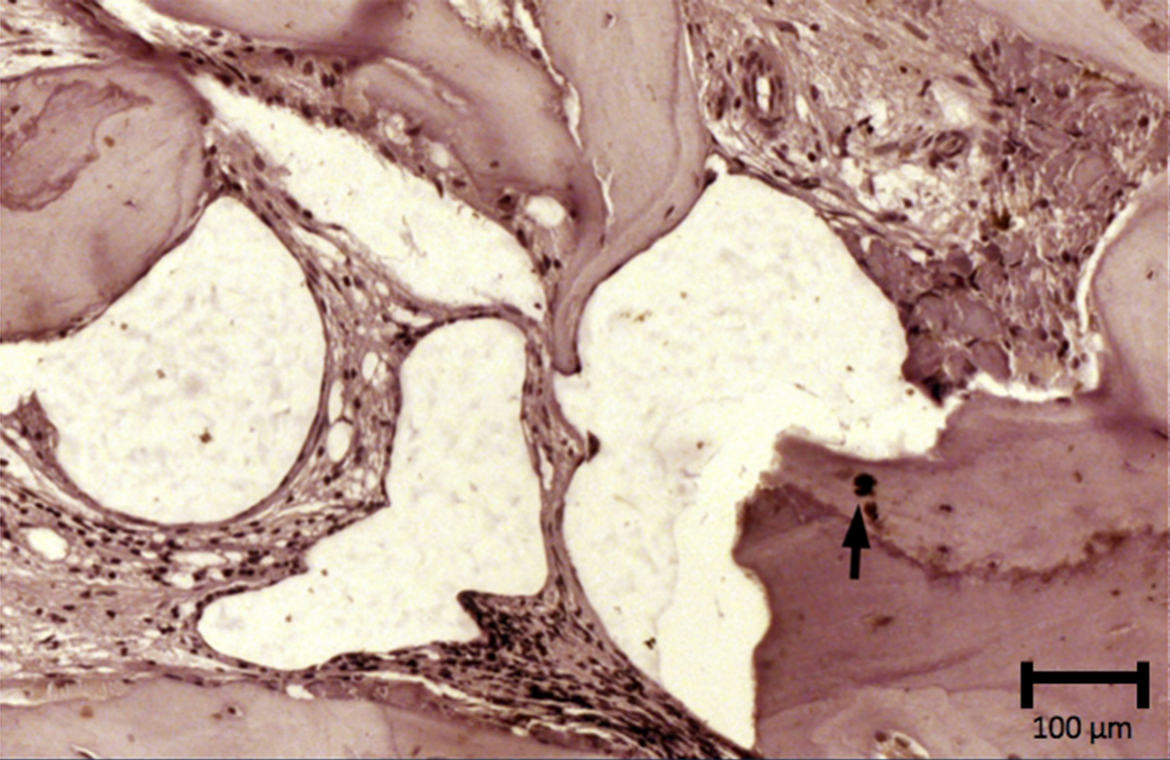
Histological section showing positive immunolabeling in cells for TRAP (→) in group 2. Harri’s hematoxylin stain, x25 magnification

A single evaluator assigned the scores for the protein probed in the two groups. For TRAP, a low (“1”) level of osteoclast-specific staining presented in both groups, indicated that these two biomaterials were in a remodeling phase (Figure 5). This protein stained multinucleated cells on bone surface or on bone periphery (Figures 6 and 7).

### Volumetric analysis

**Figure 8:**
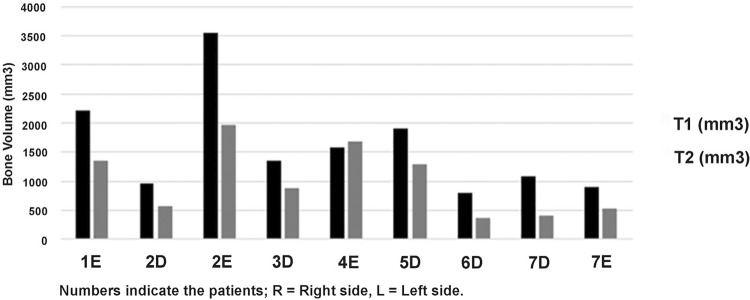
Graphic demonstrating the bone volume grafted (mm3) into maxillary sinus after 15 days (T1) and after six months of bone healing (T2) in group 1

**Figure 9:**
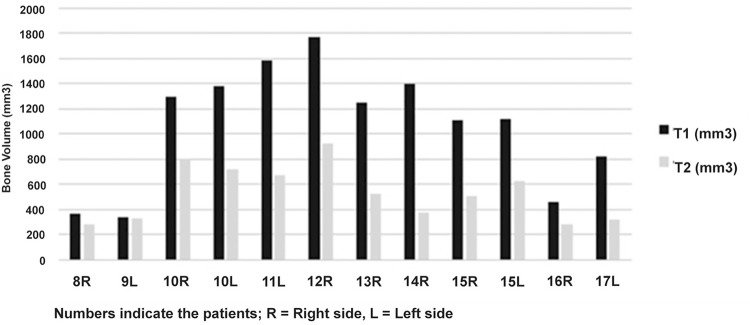
Graphic demonstrating the bone volume grafted (mm3) into maxillary sinus after 15 days (T1) and after six months of bone healing (T2) in group 2

**Figure 10:**
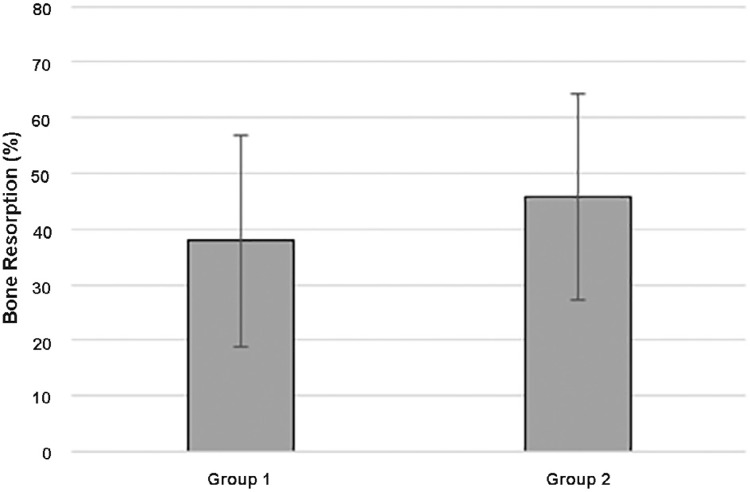
Graphic demonstrating the average of bone graft resorption in groups 1 and 2

All the volumes were calculated in mm ^3^ . The bone volume change rate ranged from 32.4% to 53.2% (37.9%±18.9) for group 1; however, one case presented an increase of 5.8% of the bone volume (Figure 8). Group 2 presented rates that ranged from 3.3% to 73.6% (45.7%±18.5) (Figure 9). No statistical difference was found between the groups (P=0.82) (Figure 10). The correlation coefficient for the volume changes of the augmented bone and the elapsed time was r=0.88 for group 1 and r=0.82 for group 2, which indicates a progressive bone resorption.

## Discussion

Characteristics such as bone quality and quantity are fundamental when oral rehabilitations with osseointegrated implants are planned. In Dentistry, particulate biomaterials are excellent alternatives for bone volume restoration ^24^ . To promote a satisfactory biological interaction, in which the biomaterial is biocompatible, allowing the formation and maintenance of bone volume, it is important that these biomaterials provide favorable chemical and mechanical properties.

Bioactive glass is an osteoconductive biomaterial capable to bind with the new bone formed by chemical adhesion ^28^ . This phenomenon occurs through a corrosion of the glass surface by the tissue fluids, promoting a layer of calcium rich in phosphorus and a sublayer with silica that adhere tightly to the apatite crystals of the bone ^6^ . Due to these features, studies have shown promising results using this material in periodontal defects and in maxillary sinus bone augmentation ^4^ ^,^ ^5^ ^,^ ^25^ ^-^ ^27^ .

Tadjoedin, et al. ^25^ (2000) proposed a split mouth study comparing the autogenous bone graft with bioactive glass alone in the human maxillary sinus. This study was performed with only three patients, harvesting the samples in three periods (after four months, after six months, and after 15 months). The study found that bone formation improved 26.9% after four months and 35.6% after six months. However, it was considered a pilot study due to the small number of patients. In this study, after six months of bone repair, the bioactive glass group presented areas of woven bone with type IV typical trabecular bone and well-cellularized connective tissue. The autogenous group presented a lamellar matrix with low areas of immature bone and the presence of osteoblasts in the periphery. The authors observed a similar rate of bone formation, which was slower when the biomaterial was used.

Cordioli, et al. ^5^ (2001) evaluated the bioactive glass added to autogenous bone graft in a 4:1 ratio in human maxillary sinus height reconstruction. In their research they did not compare this bone substitute against a control group. The biopsies were evaluated in two regions, coronal and apical orientation with 30.6% and 14.2% of new bone formation, respectively. In comparison, the outcomes of this study, the mixture of both biomaterials in a 1:1 ratio has 6% more bone formation in coronal region and 31.6% in apical region.

Cosso, et al. ^6^ (2000) used the bioactive glass associated with maxillary sinus lift for posterior dental implant installation. During the implant placement, bone samples were obtained with a trephine. After histological analysis, these authors found evidence of new bone formation between the particles of the material and, in some cases, inside the particles, and they suggested that this biomaterial could be used alone or associated with autogenous graft due to its high osteoconductive potential. In our study, new bone formation with lamellar characteristics was observed in the pristine bone, intermediate, and apical regions in both groups. These findings after six months of maxillary sinus augmentation indicate that bioactive glass added to an autogenous bone graft has the same behavior of the autogenous bone graft by itself.

Previous studies with bioactive glass did not respond to its cells behavior during the healing period in the maxillary sinuses augmentation ^5^ ^,^ ^25^ ^,^ ^27^ . In this study, the results of immunolabeling for TRAP after six months in the group 1 demonstrated osteoclast activity in the new bone formed, suggesting a period of bone remodeling.

Johansson, et al. ^9^ (2001) also used computed tomography (CT) analysis to evaluate the autogenous bone resorption in patients with severely atrophic edentulous maxilla treated with onlay grafts and particulate bone grafts for maxillary sinus lifting. CT scans were obtained in the first two weeks postoperatively and after 6–7 months. At this time, the volumes of the inlay and onlay grafts were reduced on average by 49.5% and 47.0% of the initial volume, respectively. These results agree with those obtained in this study, since there was a resorption mean of 45.7% in the group in which autogenous bone alone was used.

In 2015, Gorla, et al. ^8^ (2015) compared the reabsorption of pure beta-tricalcium phosphate (β-TCP), β-TCP added to autogenous bone in a 1:1 ratio and autogenous bone graft alone in human maxillary sinuses bone augmentation. Their results demonstrated that the pure β-TCP and its mixture with autogenous bone graft presented similar volumetric behavior compared with the autogenous bone graft alone after six months of bone repair. These outcomes corroborate this study in which bioactive glass mixed with autogenous bone graft 1:1 has similarity with autogenous bone graft alone.

## Conclusions

In conclusion, this study demonstrated that the bioactive glass added with autogenous bone graft 1:1 presents similar volumetric shrinkage, new bone formation, and osteoclastic activity when compared with autogenous bone graft only.
